# No differences in knee anthropometric‐related risk factors between unilateral and bilateral ACL reconstruction: A matched MRI‐based cohort study

**DOI:** 10.1002/jeo2.70394

**Published:** 2025-08-05

**Authors:** Riccardo D'Ambrosi, Fabrizio Di Maria, Luca Maria Sconfienza, Stefano Fusco, Maria Vittoria Bausano, Mattia Sica, Francesco Silletta, Elisabeth Abermann, Christian Fink

**Affiliations:** ^1^ IRCCS Istituto Ortopedico Galeazzi Milan Italy; ^2^ Dipartimento di Scienze Biomediche per la Salute Università degli Studi di Milano Milan Italy; ^3^ Department of General Surgery and Medical Surgical Specialties, Section of Orthopaedics and Traumatology University Hospital Policlinico “Rodolico‐San Marco” University of Catania Catania Italy; ^4^ Scuola di Specializzazione in Radiodiagnostica Università degli Studi di Milano Milan Italy; ^5^ Scuola di Specializzazione in Radiodiagnostica Università degli Studi di Salerno Salerno Italy; ^6^ Gelenkpunkt‐Sports and Joint Surgery FIFA Medical Centre of Excellence Innsbruck Austria; ^7^ Research Unit for Orthopaedic Sports Medicine and Injury Prevention (OSMI) Private University for Health Sciences Medical Informatics and Technology Innsbruck Austria

**Keywords:** anterior cruciate ligament, bilateral, lateral femoral condyle ratio, notch index, posterior tibial slope, risk factor

## Abstract

**Purpose:**

The primary aims of this retrospective study were to (1) compare medial posterior tibial slope (mPTS), lateral posterior tibial slope (lPTS), notch index and lateral femoral condyle ratio (LFCR) between patients who have undergone unilateral versus bilateral anterior cruciate ligament reconstruction (ACLR), measured on magnetic resonance imaging (MRI) using a matched cohort analysis; (2) evaluate whether subgroup differences exist based on age, gender and side; (3) assess risks factors for ACL injury using logistic models.

**Methods:**

This retrospective study included patients who underwent primary ACLR between 2015 and 2019. Measurements of the unilaterally operated knee (*n *= 45) were matched using propensity score‐matched in a ratio of 1:1 with the corresponding knee in the bilateral group (*n* = 45) based on age, sex, side, using the greedy nearest neighbour method. Exclusion criteria included inadequate MRI quality (<1.5 Tesla), concomitant ligament injuries or fractures, and <6‐year follow‐up for unilateral ACLR patients. Five blinded reviewers measured mPTS, lPTS, femoral notch index and LFCR on MRI scans.

**Results:**

No significant differences were observed between the bilateral and unilateral groups for mPTS, lPTS, femoral notch index or LFCR. The mean values for the bilateral group were: mPTS, 3.84° ± 2.54°; lPTS, 6.03° ± 3.63°; notch index, 0.27 ± 0.02; and LFCR, 0.73 ± 0.07. Corresponding values for the unilateral group were: mPTS, 3.92° ± 2.94°; lPTS, 6.37° ± 3.13°; notch index, 0.27 ± 0.03; and LFCR, 0.71 ± 0.06 (all *p* > 0.05). Subgroup analysis revealed a statistically significant difference only for the femoral notch index in patients older than 25 years: bilateral ACLR (0.29 ± 0.03) versus unilateral ACLR (0.27 ± 0.03; *p* = 0.027).

**Conclusions:**

Patients who underwent bilateral ACLR showed no significant differences in mPTS, lPTS, femoral notch index or LFCR, compared with those who underwent unilateral ACLR, irrespective of age, gender and side.

**Level of Evidence:**

Level III, cross‐sectional study.

**Abbreviations**
ACL: anterior cruciate ligament
ACLR: anterior cruciate ligament reconstruction
CI: confidence interval
lPTS: lateral posterior tibial slope
LFCR: lateral femoral condyle ratio
mPTS: medial posterior tibial slope
MRI: magnetic resonance imaging
MTPD: medial tibial plateau depth
PACS: Picture Archiving and Communication System
PTS: posterior tibial slope
STROBE: Strengthening the Reporting of Observational Studies in Epidemiology

## INTRODUCTION

Anterior cruciate ligament (ACL) tears are among the most common orthopaedic injuries, accounting for approximately 50% of all knee injuries, with an annual incidence of one in every 3500 individuals in the United States [3]. Patient‐specific factors influencing the risk of ACL tears, such as age, biological sex and activity level, have been extensively studied. Prior research has indicated that ACL ruptures are most prevalent among younger individuals, particularly adolescents and those in their twenties [5, 26, 31]. The incidence of nonsimultaneous bilateral ACL injuries ranges from 1.1% to 14% of all ACL injuries, whereas simultaneous ruptures are exceedingly rare and primarily documented as isolated case reports [13]. The risk of sustaining the contralateral knee injuries exceeds the risk of primary rupture, with subsequent injuries most often occurring within the first 3 years following initial surgery [20, 37]. Despite numerous theories, the causes of bilateral ACL injuries remain inadequately understood. Anthropometric, biomechanical, neuromuscular, anatomical, hormonal and genetic factors are considered specialized internal risk factors and are the subject of ongoing research [9]. Narrowing of the intercondylar notch, first identified by Palmer in 1936 as a potential risk factor for ACL injury, has been supported by some studies, while others have found no significant correlation. Few studies have explored the relationship between bilateral ACL injuries and the morphology of the intercondylar notch [2, 30, 35]. The bony anatomy of the lateral femoral compartment has also garnered significant attention for its role in pivot shift mechanics. Recent evidence suggests that the lateral femoral condyle ratio (LFCR), as measured on radiographs, may predict noncontact ACL injuries. However, it remains unclear whether LFCR, when assessed via magnetic resonance imaging (MRI), is a risk factor for noncontact ACL injuries [15, 24]. In addition, an increased posterior tibial slope (PTS) has been associated with a higher frequency of ACL rupture. Cadaveric studies suggest up to 7° difference between the medial posterior tibial slope (mPTS) and lateral posterior tibial slope (lPTS) within a single knee [6]. Recent research proposes that the medial and lPTS should be evaluated separately, as individuals with ACL ruptures often exhibit a steeper slope on the lateral plateau [4, 11, 23]. To date, no studies have compared patients with a history of bilateral versus unilateral anterior cruciate ligament reconstruction (ACLR) using several different measurements on MRI, including mPTS, lPTS, notch index and LFCR, while also performing subgroup analyses by side, age and gender.

The primary aims of this retrospective study were to (1) compare mPTS, lPTS, notch index and LFCR between patients who have undergone unilateral versus bilateral ACLR, measured on MRI using a matched cohort analysis; (2) evaluate whether subgroup differences exist based on age, gender and side; (3) assess risks factors for ACL injury using logistic models.

## MATERIALS AND METHODS

This study was conducted in accordance with the Strengthening the Reporting of Observational Studies in Epidemiology (STROBE) declaration [7]. All procedures were conducted in compliance with the principles outlined in the 1964 Declaration of Helsinki and its subsequent amendments [38].

The study protocol was approved by our institutional review board (protocol number ACL‐L2104). Data and demographic information were retrospectively collected from all patients who underwent ACLR between 2015 and 2018. All surgeries were performed by the senior author (C. F.).

Patients were eligible for inclusion if they were skeletally mature, aged between 18 and 75 years, and have undergone primary nonsimultaneous bilateral ACLR or primary unilateral ACLR, with adequate imaging defined as MRI at a minimum of 1.5 T.

**Figure 1:**
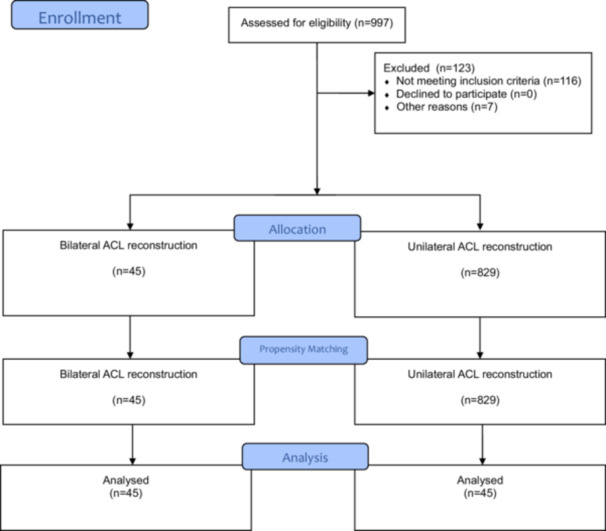
Flow chart of patient enrolment. ACL, anterior cruciate ligament.

Patients were excluded if they had a prior surgical history on the index knee, concomitant injuries including tibial or femoral fractures, complete tears of the posterior cruciate ligament, quadriceps or patellar tendon, intra‐articular infections or malignancies. Additionally, patients with unilateral ACLR who had less than 6 years of clinical follow‐up were further excluded from the study. The 6‐year minimum follow‐up threshold was applied to ensure that any subsequent contralateral ACL injuries, which may not have occurred in patients with shorter follow‐up, were adequately considered [13]. This longer follow‐up period strengthens the comparisons between unilateral and bilateral ACLR groups. Measurements of the unilaterally operated knee were matched were propensity score‐matched in a ratio of 1:1 with the corresponding knee in the bilateral group based on age, sex, side, using the greedy nearest neighbour method [21]. A flow diagram for the inclusion of analyzed patients is demonstrated in Figure 1 [1].

All measurements were performed independently by five readers, including two board‐certified musculoskeletal radiologists (L. M. S. and S. F.) and three radiology residents (M. V. B., M. S. and F. S.). All readers were blinded to the clinical history of the patients. Images were accessed via the picture archiving and communication system (PACS), and all measurements were conducted using our institution′s PACS workstation (Visage Imaging, Sectra IDS7).

### MRI protocol

All MRI scans were performed on scanners with a field strength ≥ 1.5 T. Each examination included a minimum of four sequences (with slice thickness ≤ 3 mm), consisting of fat‐suppressed proton‐density or intermediate‐weighted sequences in the axial, sagittal and coronal planes, as well as sagittal T1‐weighted images.

### Femoral notch width index

**Figure 2:**
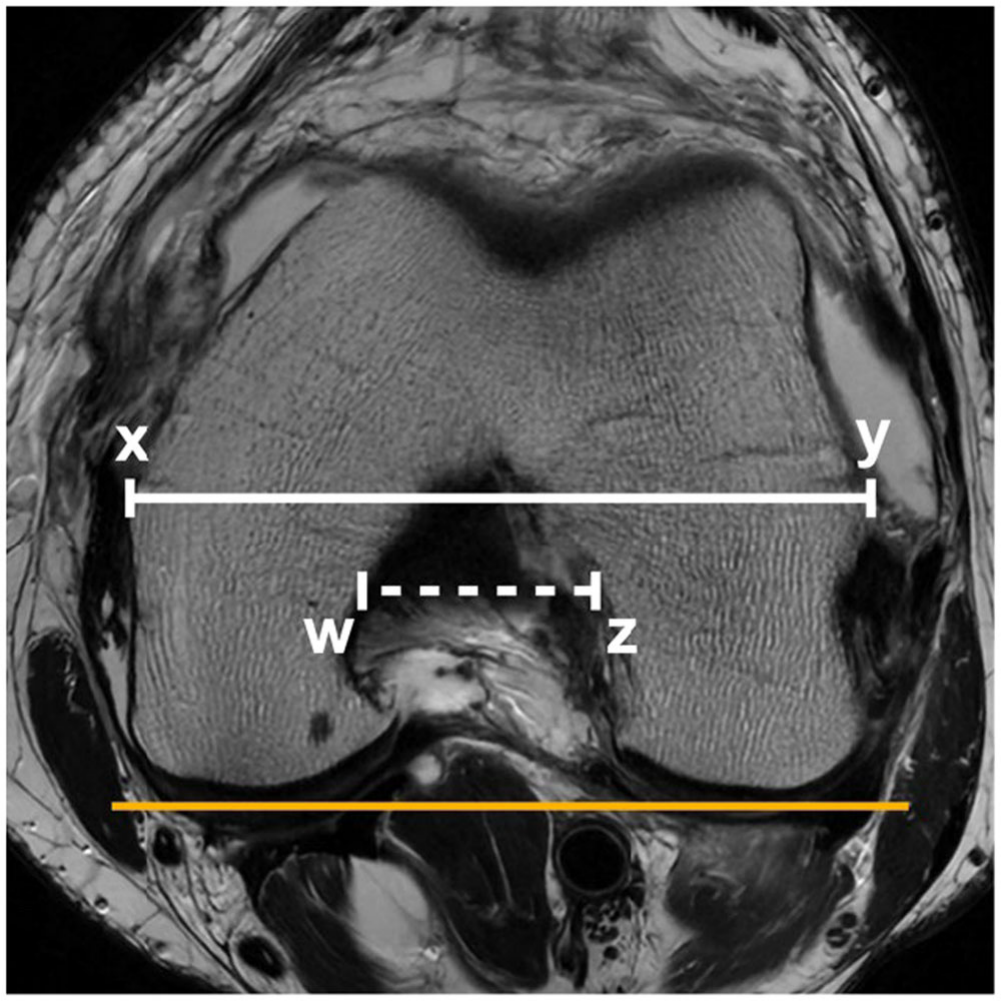
Measurement of notch width index on axial T2w image. The transcondylar width (xy) is divided by the femoral notch width (wz). Both lines are parallel to a line tangent to the posterior surface of the femoral condyles (orange line).

The femoral notch width index was calculated by dividing the transcondylar width by the femoral notch width. Measurements were performed on axial MRI images at the level of the popliteal groove. The notch width consisted of the length between the medial profile of the lateral condyle and the lateral profile of the medial condyle of the femur. The transcondylar width was defined as the distance between the outermost points of the femoral condyles. Both lines were parallel to a line tangent to the posterior surface of the femoral condyles [19] (Figure 2).

### LFCR

**Figure 3:**
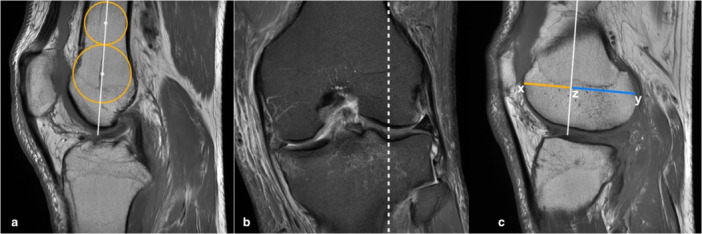
Measurement of the lateral femoral condyle ratio. (a) On the central sagittal image, two circles are drawn at the centre of the femur shaft to obtain the long axis of the distal femur (white line passing through the centres of the circles). (b) On the coronal plane, the central point of the lateral femoral condyle is identified (dotted line). (c) On the sagittal image passing for the dotted line in (b), the long axis of the distal femur is replicated. A perpendicular line to the femoral axis is drawn between the most anterior and posterior points of the lateral condyle (xy). The distance between the intersection of the two lines to the posterior profile of the condyle (zy) is divided by the total length of the condyle (xy) to obtain the LFCR. (LFCR = zy/xy).

The first step for the measurement of the LFCR was to select the central sagittal T1 image by identifying the slice passing through the tibial attachment of the posterior cruciate ligament and the intercondylar eminence, where the anterior and posterior tibial cortices appeared in a concave shape. Two circles were drawn at the centre of the femur shaft, touching the anterior and posterior femoral cortex. The more distant circle was placed at the distal aspect of the shaft. A line passing through the centres of the two circles was considered to be the long axis of the distal femoral (Figure 3a). Second, the central point of the lateral femoral condyle was identified on the coronal plane (Figure 3b). On the corresponding sagittal T1 image, the line representing the long axis of the distal femur was replicated; a perpendicular line to the femoral axis was drawn between the most anterior and posterior points of the lateral condyle (Figure 3c). The distance from the intersection of these two lines to the posterior profile of the condyle was divided by the total length of the condyle and multiplied by 100%. This ratio was defined as the LFCR [15].

### Medial and lateral tibial slope

**Figure 4:**
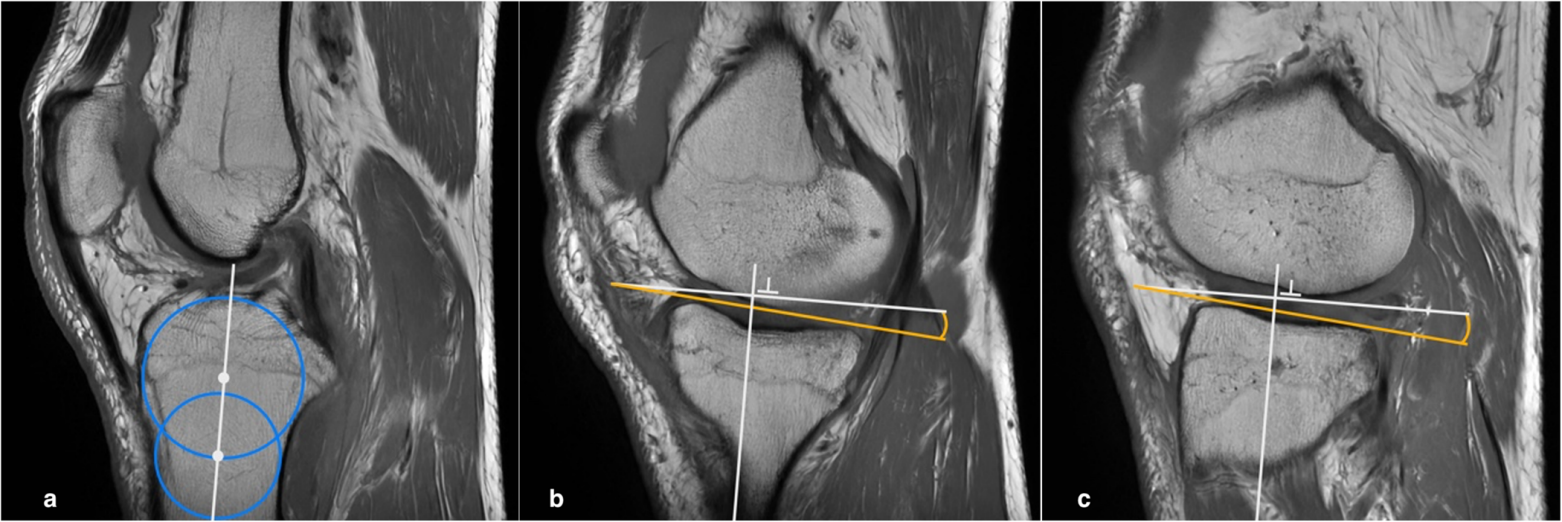
Assessment of tibial slope. (a) On the central sagittal image, two circles are drawn at proximal tibia to obtain the tibial longitudinal axis (white line passing through the centres of the circles). (b, c) Sagittal images passing for the mediolateral point of the medial (b) and lateral (c) tibial plateau. The tibial slope represents the angle formed by the orthogonal line to the longitudinal axis (white horizontal line) and the tangent line (orange line) to the superior profile of the medial (b) and lateral (c) tibial plateau.

On the central sagittal T1 image (same slice selected in the step 1 for the measurement of LFCR), two circles were placed in the proximal tibia. The cranial circle had to touch the anterior, posterior and cranial tibial cortex bone and the caudal circle had to touch the anterior and posterior cortex border. In cases with vague borders between the cortex and the medullary canal, the middle of the transition zone between a definitive black cortex and a light grey medullary canal was chosen. The centre of the caudal circle was positioned on the circumference of the cranial circle to standardize the distance between the circles. The tibial longitudinal axis was defined by a line passing through the centres of these two circles (Figure 4a). The last step consisted of identifying the sagittal image at the mediolateral centre of the medial plateau, using the coronal or axial images as reference. On this image, a tangent to the medial plateau connecting the uppermost superior–anterior and posterior cortex edges was drawn. The slope of the medial plateau was defined by the angle between the tangent to the medial plateau and the perpendicular line to the tibial longitudinal axis (Figure 4b). The same process was made at the mediolateral central of the lateral plateau, to determine the lateral tibial slope [17] (Figure 4c).

### Statistical analysis

Sample characteristics and scores (femoral notch index, LFCR, medial and lateral tibial slope) were presented by groups using absolute frequencies and percentages or mean ± standard deviation. Continuous variables were assessed for normality with Shapiro–Wilks test. Spearman rank correlations were computed overall and within each group to explore relationships among the collected variables. Subgroup analyses were conducted to compare groups within subjects grouped by sex, age or knee side. Age was dichotomized at the overall median value for analysis. Additionally, differences by sex, age and side were examined within knees of the same group. Statistical differences were evaluated using *χ* ^2^ test or Fisher′s exact test for categorical variables, and *t* test or Wilcoxon–Whitney test for continuous variables, depending on the distribution of scores.

Linear mixed models, or logistic mixed models (depending on the analyzed variables), were performed with a random intercept for each subject to account for the paired structure of the data. Scores were dichotomized at their mean value in the population using the following cut‐offs, based on previous studies: 4.6° for medial tibial slope, 5° for lateral tibial slope, 0.28 for femoral notch index and 0.6 for LFCR [15, 17, 18]. Knees were classified as at higher risk of rupture if they had a medial or lateral tibial slope angle and LFCR above the cut‐off, or a femoral notch index below the cut‐off. Differences between groups were tested using *χ* ^2^ or Fisher′s exact tests as appropriate.

To assess variables associated with knees at higher risk of rupture, multivariate logistic models were created for each dichotomized score with the following independent variables: group, age, gender, side and scores differing from the dependent variables. To control for the paired structure of the data, the same multivariate models were estimated with random intercepts for each subject (i.e., multivariate logistic mixed models). A two‐tailed *p*‐value of <0.05 was considered statistically significant. All statistical analyses were performed using R version 4.3.0 (R Foundation for Statistical Computing, Vienna, Austria. URL: https://www.R-project.org/).

### Sample size

The required sample size was calculated to ensure adequate power for detecting differences in key outcomes between groups. A sample of 90 knees (45 per group) was determined to provide 80% power to detect a mean difference of 1.5 points in the femoral notch index between groups, assuming a standard deviation of 2.5 in each group, using a two‐tailed *t*‐test with a 5% alpha error. For the LFCR, the same sample size provided 80% power to detect a mean difference of 0.03 points, assuming a standard deviation of 0.05 in each group. Similarly, the sample size allowed for detecting a mean difference of 2° in the medial tibial slope angle, with an assumed standard deviation of 3° in each group, and a mean difference of 2° in the lateral tibial slope angle, with an assumed standard deviation of 3.5° in each group [17].

## RESULTS

A retrospective review identified 997 patients who underwent unilateral ACL reconstruction (ACLR) with a minimum follow‐up of 6 years, along with 82 patients who underwent bilateral ACLR. After applying exclusion criteria, 836 patients with unilateral ACLR and 45 patients with bilateral ACLR remained eligible for propensity score matching. A 1:1 matching process was conducted, resulting in a final cohort of 90 patients (*n* = 90), with 45 in the bilateral ACLR group and 45 in the unilateral ACLR group.

Balance diagnostics following propensity score matching demonstrated a 99.9% reduction in the standardized mean difference of the logit propensity score and a variance ratio of 0.998.

### Demographic data

A total of 90 patients were included in the study, equally distributed between the bilateral and unilateral ACLR groups (45 patients each). Among these, 22 females were in the bilateral ACLR group and 23 in unilateral ACLR group (*p* > 0.99). Both groups included 27 left knees (*p* > 0.99). The mean age in the bilateral ACLR group was 26.40 ± 10.82 years, compared with 30.36 ± 12.74 in the unilateral ACLR group (*p* = 0.075). No re‐ruptures were reported during all the follow‐up periods.

Detailed data are reported in Table 1.

**Table 1:** Demographic data of the patients included in the study.

	Bilateral	Unilateral		
	*N* = 45	*N* = 45		
	*n* (%)	*n* (%)	*p* value	Adj *p* valu*e* a
Sex				
Female	22 (48.9)	23 (51.1)	>0.99	0.586
Male	23 (51.1)	22 (48.9)		
Knee side				
Left	27 (60.0)	27 (60.0)	>0.99	>0.99
Right	18 (40.0)	18 (40.0)		

	**Mean ± SD;**	Mean ± SD;		
	Median [IQR]	Median [IQR]		
Age (years)	26.40 ± 10.82	30.36 ± 12.74	0.075	0.196
	23.00 [19.00, 29.00]	27.00 [21.00, 42.00]		
Age range				
≤25 years	27 (60.0)	20 (44.4)	0.205	0.273
>25 years	18 (40.0)	25 (55.6)		

*Note*: *p* < 0.05 = statistically significant difference.

^a^ Analysis adjusted for the paired structure of data.

### Bilateral versus unilateral radiologic measurements

Radiological score revealed no significant differences between the bilateral and unilateral ACLR groups for any of the measured parameters. In the bilateral ACLR group, mPTS, lPTS, notch index and LFCR were 3.84° ± 2.54°, 6.03° ± 3.63°, 0.27 ± 0.02, and 0.73 ± 0.07, respectively. In the unilateral ACLR group, the corresponding values were 3.92° ± 2.94°, 6.37 ± 3.13, 0.27 ± 0.03 and 0.71 ± 0.06. None of these differences reached statistical significance (all *p* > 0.05). Detailed results are reported in Table 2.

**Table 2:** Radiological analysis between patients with bilateral versus unilateral anterior cruciate ligament reconstruction.

	Bilateral	Unilateral		
	*N* = 45	*N* = 45		
	Mean ± SD	Mean ± SD		
	Median [IQR]	Median [IQR]	*p* value	Adj *p* value
Medial tibial slope angle (degrees)	3.84 ± 2.54;	3.92 ± 2.94;	0.920	0.983
	3.40 [2.20, 4.80]	3.60 [1.40, 5.90]		
Lateral tibial slope angle (degrees)	6.03 ± 3.63	6.37 ± 3.13	0.485	0.959
	4.90 [2.90, 8.70]	6.20 [3.60, 8.90]		
Notch index	0.27 ± 0.02	0.27 ± 0.03	0.280	0.495
	0.27 [0.26, 0.29]	0.27 [0.25, 0.28]		
Lateral femoral condyle ratio	0.73 ± 0.07;	0.71 ± 0.06;	0.212	0.467
	0.73 [0.67, 0.79]	0.72 [0.67, 0.76]		

*Note*: Analysis adjusted for the paired structure of data. *p* < 0.05 = statistically significant difference.

### Correlations

Significant correlations were observed only within the bilateral ACLR group. Specifically, a positive correlation was found between the mPTS and lPTS (correlation = 0.35; *p* = 0.02). Additionally, a negative correlation was identified between mPTS and LFCR (correlation = 0.36; *p* = 0.016). Detailed results are reported in Table 3.

**Table 3:** Analysis of correlations between patients with bilateral versus unilateral anterior cruciate ligament reconstruction.

		Overall (*N* = 90)		Bilateral (*N* = 45)		Unilateral (*N* = 45)	
Variables		Correlation	*p* value	Correlation	*p* value	Correlation	*p* value
Age	Medial tibial slope angle (degrees)	−0.16	0.135	−0.16	0.307	−0.15	0.317
Age	Lateral tibial slope angle (degrees)	−0.06	0.573	0.05	0.755	−0.19	0.210
Medial tibial slope angle (degrees)	Lateral tibial slope angle (degrees)	0.31	0.003*	0.35	0.020*	0.27	0.073
Age	Notch index	0.13	0.216	0.27	0.072	0.04	0.792
Medial tibial slope angle (degrees)	Notch index	0.07	0.514	0.00	0.988	0.12	0.443
Lateral tibial slope angle (degrees)	Notch index	−0.08	0.463	−0.07	0.641	−0.13	0.396
Age	Lateral femoral condyle ratio	0.02	0.830	0.15	0.330	−0.08	0.612
Medial tibial slope angle (degrees)	Lateral femoral condyle ratio	−0.31	0.003*	−0.36	0.016*	−0.29	0.051
Lateral tibial slope angle (degrees)	Lateral femoral condyle ratio	−0.06	0.556	−0.05	0.750	−0.03	0.834
Notch index	Lateral femoral condyle ratio	−0.03	0.761	−0.07	0.640	−0.02	0.897

*Note*: *p* < 0.05 = statistically significant difference.

* Statistical significant difference.

### Subgroups analysis

Subgroup analysis by age, gender and knee side revealed a single statistically significant difference. Among patients older than 25 years, the femoral notch was higher in the bilateral ACLR group (0.29 ± 0.03) compared with the unilateral ACLR group (0.27 ± 0.03) (*p* = 0.027). Detailed results are reported in Table 4.

**Table 4:** Analysis of subgroups between patients with bilateral versus unilateral anterior cruciate ligament reconstruction.

	Bilateral	Unilateral		
	Mean ± SD	Mean ± SD	*p* value	Adj *p* value^a^
Male	*N* = 23	*N* = 22		
Medial tibial slope angle (degrees)	3.60 ± 2.84	3.73 ± 2.93	0.892	0.966
Lateral tibial slope angle (degrees)	5.90 ± 3.43	7.39 ± 2.96	0.128	0.336
Notch index	0.27 ± 0.02	0.26 ± 0.03	0.404	0.543
Lateral femoral condyle ratio	0.72 ± 0.06	0.70 ± 0.07	0.371	0.540
Female	*N* = 22	*N* = 23		
Medial tibial slope angle (degrees)	4.08 ± 2.22	4.11 ± 3.01	0.733	0.954
Lateral tibial slope angle (degrees)	6.16 ± 3.91	5.40 ± 3.05	0.650	0.354
Notch index	0.28 ± 0.03	0.27 ± 0.02	0.480	0.671
Lateral femoral condyle ratio	0.74 ± 0.07	0.72 ± 0.06	0.359	0.560
Age > 25 years	*N* = 18	*N* = 25		
Medial tibial slope angle (degrees)	3.96 ± 2.10	3.50 ± 2.81	0.295	0.923
Lateral tibial slope angle (degrees)	6.14 ± 3.43	6.05 ± 3.13	0.971	0.905
Notch index	0.29 ± 0.03	0.27 ± 0.03	0.027*	0.062
Lateral femoral condyle ratio	0.73 ± 0.07	0.71 ± 0.07	0.227	0.370
Age ≤ 25 years	*N* = 27	*N* = 20		
Medial tibial slope angle (degrees)	3.76 ± 2.84	4.46 ± 3.10	0.451	0.636
Lateral tibial slope angle (degrees)	5.96 ± 3.83	6.76 ± 3.17	0.322	0.747
Notch index	0.26 ± 0.02	0.27 ± 0.02	0.507	0.437
Lateral femoral condyle ratio	0.73 ± 0.07	0.72 ± 0.06	0.648	0.882

*Note*: *p* < 0.05 = statistically significant difference.

^a^ Analysis adjusted for the paired structure of data. No adjustment was needed for the analysis within knees from the same side.

* Statistical significant difference.

### Logistic regression and odds ratio

After adjusting for other variables: lateral tibial slope was positively associated with elevated medial tibial slope angle values. The adjusted odds ratio for each 1° increase in the lateral tibial slope was 1.26 (95% confidence interval [CI]: 1.04–1.52, *p* = 0.017).

A higher LFCR was positively associated with elevated medial tibial slope angle values. The adjusted odds ratio for each 1° increase in the LFCR was 0.29 (95% CI: 0.1–0.85, *p* = 0.024). Detailed results are reported in Table 5.

**Table 5:** Logistic regression between patients with bilateral versus unilateral anterior cruciate ligament reconstruction.

	Lower rupture risk	Higher rupture risk^a^	Adjusted model^c^ AdjOR (95% CI, *p* value)
Dependent:Medial tibial slope angle	4.6 ≤ degrees	>4.6 degrees	
Type			
Bilateral	29 (64.4)	16 (35.6)	Ref. category
Unilateral	28 (62.2)	17 (37.8)	0.98 (0.33–2.93; *p *= 0.970)
Lateral tibial slope angle (degrees)	5.4 ± 3.3	7.5 ± 3.1	1.26 (1.04–1.52; *p* = 0.017 *)
Notch index b	27.0 ± 2.4	27.1 ± 2.7	1.02 (0.82–1.28; *p* = 0.835)
Lateral femoral condyle ratio d	7.3 ± 0.6	7.0 ± 0.6	0.29 (0.1–0.85; *p* = 0.024 *)
Age	29.7 ± 11.9	26.1 ± 11.7	0.97 (0.92–1.02; *p* = 0.225)
Gender			
Female	27 (60.0)	18 (40.0)	Ref. category
Male	30 (66.7)	15 (33.3)	0.41 (0.12–1.4; *p* = 0.155)
Side			
Left	33 (61.1)	21 (38.9)	Ref. category
Right	24 (66.7)	12 (33.3)	0.6 (0.2–1.81; *p* = 0.367)

**Dependent: Lateral tibial slope angle**	**≤5 degrees**	**>5 degrees**	
Type			
Bilateral	23 (51.1)	22 (48.9)	Ref. category
Unilateral	17 (37.8)	28 (62.2)	2.52 (0.57–11.21; *p* = 0.225)
Medial tibial slope angle (degrees)	3.0 ± 2.3	4.5 ± 2.9	1.46 (0.99–2.16; *p* = 0.054)
Notch index c	27.2 ± 2.2	26.9 ± 2.7	0.95 (0.72–1.25; *p* = 0.695)
Lateral femoral condyle ratio d	7.3 ± 0.7	7.2 ± 0.6	1.55 (0.48–4.94; *p* = 0.462)
Age	29.3 ± 12.1	27.6 ± 11.8	0.98 (0.92–1.04; *p* = 0.564)
Gender			
Female	24 (53.3)	21 (46.7)	Ref. category
Male	16 (35.6)	29 (64.4)	5.39 (0.6‐48.42; *p* = 0.133)
Side			
Left	24 (44.4)	30 (55.6)	Ref. category
Right	16 (44.4)	20 (55.6)	1.38 (0.4–4.78; *p* = 0.615)

**Dependent: Notch index**	**>0.28**	**≤0.28**	
Type			
Bilateral	17 (37.8)	28 (62.2)	Ref. category
Unilateral	13 (28.9)	32 (71.1)	2.33 (0.51–10.55; *p *= 0.272)
Lateral tibial slope angle (degrees)	5.9 ± 3.2	6.3 ± 3.5	1.07 (0.85–1.34; *p* = 0.571)
Medial tibial slope angle (degrees)	4.0 ± 2.5	3.8 ± 2.9	0.9 (0.66–1.21; *p* = 0.475)
Lateral femoral condyle ratio d	7.2 ± 0.7	7.2 ± 0.6	1.02 (0.33–3.17; *p *= 0.969)
Age	31.8 ± 12.1	26.7 ± 11.6	0.94 (0.86–1.02; *p* = 0.128)
Gender			
Female	17 (37.8)	28 (62.2)	Ref. category
Male	13 (28.9)	32 (71.1)	1.63 (0.38–7.01; *p* = 0.510)
Side			
Left	16 (29.6)	38 (70.4)	Ref. category
Right	14 (38.9)	22 (61.1)	0.6 (0.17–2.09; *p* = 0.420)

**Dependent: Lateral femoral condyle ratio**	**≤0.6**	**>0.6**	
Lateral tibial slope angle (degrees)	7.0 ± 5.4	6.2 ± 3.4	0.93 (0.61–1.43; *p* = 0.737)
Medial tibial slope angle (degrees)	4.0 ± 3.5	3.9 ± 2.7	1.08 (0.63–1.88; *p* = 0.771)
Notch index c	26.8 ± 2.2	27.0 ± 2.5	0.97 (0.48–1.95; *p* = 0.931)
Age	21.0 ± 15.6	28.5 ± 11.9	1.1 (0.88–1.38; *p* = 0.406)

*Note*: *p* < 0.05 = statistically significant difference.

Abbreviation: CI, confidence interval.

^a^ Group of interest, coded as 1 in the logistic regression.

^b^ Results of the multiple logistic model adjusted for all the variables listed for each specific dependent variable and for the paired structure of data (i.e., for 16 subjects we included both right and left knees).

^c^ Estimates are shown for 4 an increase of 0.01 for the Notch index, and

^d^ An increase of 0.1 for the lateral femoral condyle ratio; Categorical data are reported as n (%) and continuous data as mean ± SD. *N* = 90 knees for all models.

* Statistical significant difference.

## DISCUSSION

The most significant finding of the current study is the lack of radiological differences between patients who underwent bilateral ACLR compared with those who underwent unilateral ACLR, irrespective of age, gender or side.

To our knowledge, few studies have analyzed radiological differences in bilateral ACL ruptures using diverse radiological scores, consistently highlighting the lack of significant abnormalities.

While risk factors, aetiology and mechanisms of primary ACL injuries have been widely studied, the reasons behind contralateral ACL ruptures are not clearly defined, although those injuries are not uncommon [22, 29].

The most extensive study in this area, based on the Swedish National Register of Knee Ligaments Injuries, reported that 3% of ACLR were bilateral (270 out of 9061 ACLRs). Previous studies by Souryal et al., Myklebust et al., and Fältström et al. noted bilateral injury incidences of 4%, 9% and 12%, respectively [10, 28, 34].

Motohasi analyzed patients with ACL injuries of the ACL who underwent surgery, comparing the age, activity level, injury mechanisms, surgical outcomes and radiographic findings between individuals with bilateral and unilateral injuries. All 10 patients with bilateral ACL injury were female, with 90% of these injuries resulting from noncontact mechanisms. The mean laxity score was significantly higher in the bilateral injury group compared with the unilateral injury group (*p* < 0.05). Additionally, the mean notch width index was significantly lower in the bilateral injury group than in the healthy control group (*p* < 0.05). Patients in the bilateral group also sustained their first ACL injuries at a significantly younger age than those in the unilateral group (*p* < 0.05). Furthermore, the rate of return to full sporting activity was notably lower in the bilateral group [27].

Similarly, Risitć et al. aimed to identify risk factors for bilateral ACL injury; they reported an incidence of reconstructed bilateral of 2.3% (50/2168) in relation to unilateral injuries. The study found no significant correlation between the age of the respondents or the side of the injured knee and the subjective physical activity level achieved after the second knee surgery. The average time from the first injury to surgery was 10 months, with an average interval of 4.3 years before the contralateral knee sustained an ACL injury. The majority of athletes sustained injuries during football matches. Three‐quarters of these athletes returned to competitive activities after their first operation, leading to a subsequent injury of the contralateral knee [32].

Furthermore, the prolonged absence from training and competition caused by bilateral ACL injuries can severely impact athletic performance over time, which patients often perceive as a decline in their overall quality of life [33].

In the current study, MRI was used to obtain the most precise measurements possible for medial and lateral tibial slope, notch index and LFCR. The use of MRI over radiographs has been a topic of considerable debate in the literature, with conflicting results, although MRI is predominantly supported as the preferred method [13, 18, 25].

Jahn et al. reported that tibial slope measurements using both radiography and MRI are individually reliable, the values derived from those two modalities are not interchangeable, and caution is advised when interpreting or comparing studies using these measurements [18].

Similarly, Garra et al. demonstrated that PTS measurements obtained via radiography showed

a weak to negligible correlation with those obtained using MRI [13].

Comparable results were obtained by Lin et al., who found that the mPTS measured on radiographs was significantly greater than that measured on MRI, with a low positive correlation between the two modalities [25].

In the current study, various anatomic parameters were used to assess which factors might better predict the risk of bilateral ACL injury using MRI; however, no direct correlations were found.

Biomechanical data suggest that anatomical variations in PTS can significantly impact knee stability. Several studies have reported a linear relationship between PTS and the amount of tension exerted on both native cruciate ligaments and reconstructed cruciate grafts.

Waiwaiole et al. investigated the relationship between PTS and ACL injury, aiming to determine

whether factors, such as age, race or sex correlate with ACL injury and PTS. The study found a relationship between increased lateral PTS and ACL injury, which supports the findings of previously published studies. However, medial PTS, race and sex were not identified as significant predictors of ACL injury [36].

Dean et al. conducted a systematic review of literature on PTS measurements, confirming that both lateral and medial PTS measurements were greater in patients who had failed previous ACLR, compared with those with a primary ACL tear or an intact native ACL. Additionally, the lateral PTS in patients with primary ACL tears was found to be greater than in those with an intact native ACL.

Recently, Jagadeesh et al. compared variables such as mPTS, lPTS, medial tibial plateau depth (MTPD) calculated by preoperative MRI, and posterior tibial slope calculated by lateral knee X‐ray in a cohort of patients with ACL injuries and a control group of patients without ACL injuries. The results showed that mPTS, lPTS and PTS scores were significantly higher in the ACL tear group compared with the control group (*p* < 0.01), whereas MTPD was lower in the ACL tear group compared with the control group (*p *> 0.05) [8].

Another important factor that predisposes individuals to ACL injury is the anatomy of the distal femur. Epidemiological studies of ACL‐deficient knees have identified that both female sex and a narrow femoral intercondylar notch width in both sexes are significant risk factors for ligament injury. Reduced notch width is associated with a thinner, more fragile ACL. A stenotic notch causes the ACL to impinge on the lateral femoral condyle, leading to increased anterior shear forces or tibial rotation, both of which can contribute to ACL rupture [24].

Görmeli et al. evaluated the relationship between intercondylar notch width and both unilateral and bilateral ACL injury, using magnetic resonance images. Statistically significant differences in NWI values were found between all groups, and there was a significant difference between the affected and unaffected sides in the unilateral ACL injury group.

Hoteya et al. also investigated whether a narrow intercondylar notch is associated with bilateral ACL injuries in athletes, using both MRI and radiography to assess notch size [14].

Contrary to our findings, the study by Hoteya et al. revealed that the intercondylar notch was significantly narrower in subjects with bilateral ACL injuries compared with healthy subjects.

In the last years, LFCR which has gained considerable interest due to its potential role in the phenomenon of pivot shift. He et al. investigated the effect of LFCR on the risk of noncontact ACL injuries using MRI and demonstrated that an increased LFCR is associated with a higher risk of noncontact ACL injuries [16].

Similarly, Gao et al. reported comparable findings in a study involving 72 patients who experienced ACL reruptures. They observed a significant increase in LFCR on MRI scans in patients who suffered rerupture compared with those with intact ACL reconstructions, with LFCR values of 63.38% ± 2.26% (95% CI: 62.84%–63.91%) versus 61.10% ± 2.19% (95% CI, 60.59%‐61.61%) in the intact ACLR group (*p* < 0.001) [12].

### Limitations

The current study has several limitations. As a retrospective analysis, it is inevitably subject to selection bias. However, we attempted to mitigate this bias by using random selection from a large cohort of unilateral ACLR surgeries and propensity score matching to create a reference group based on age, sex and side. Additionally, as previously noted, several measurement techniques for PTS have been developed, and these methods show varying degrees of correlation with ACL injury. The findings of this study are therefore limited to the specific techniques employed, given the ongoing debate regarding the optimal approach for evaluating PTS. Another limitation is the lack of a control group; in fact, we only included patients with ACL injury (uni‐ or bilateral) were compared, but healthy control group with no ACL injury were not assessed.

Finally, meniscal lesions were not considered as inclusion or exclusion criteria; the presence of meniscal lesions could potentially affect the measurements and outcomes, particularly the tibial slope. This factor may introduce bias in the matching process and could influence the comparison between unilateral and bilateral ACL reconstruction groups.

## CONCLUSIONS

Patients who underwent bilateral ACLR exhibited no significant differences in posterior medial and lateral tibial slope, notch index or LFCR when compared with those who underwent unilateral ACLR, irrespective of age, gender and side.

## AUTHORS CONTRIBUTIONS

**Riccardo D′Ambrosi**: Data analysis; writing manuscript; supervision; visualization; statistic; conceived the idea. **Fabrizio Di Maria**: Data analysis; statistic; writing; data curation. **Luca Maria Sconfienza**: Writing final version of the manuscript; data analysis; data curation; conceived the idea; radiologic measurement; designed the study. **Stefano Fusco**: Data curation; data analysis; radiologic measurement; writing manuscript; designed the study. **Maria Vittoria Bausano**: Data curation; data analysis; radiologic measurement; writing manuscript. **Mattia Sica**: Data curation; data analysis; radiologic measurement; writing manuscript. **Francesco Silletta**: Data curation; data analysis; radiologic measurement; writing manuscript. **Elisabeth Abermann**: Enroled and operated the patients; data analysis; writing manuscript; supervision; visualization; conceived the idea. **Christian Fink**: Enroled and operated the patients; data analysis; writing manuscript; supervision; visualization; conceived the idea; final revision of the manuscript.

## CONFLICT OF INTEREST STATEMENT

The authors declare no conflicts of interest.

## ETHICS STATEMENT

Ethical approval was appropriately obtained ACL‐L2104. All authors consent to the publication of the manuscript. All patients signed an informed to consent for the study.

## Data Availability

Raw data are available upon request to the corresponding author.
